# 425. Revealing Seasonal Patterns in the Post-Pandemic Era: A Comparative Analysis of Burden and Seasonality of Influenza and COVID-19 in Europe and the US

**DOI:** 10.1093/ofid/ofae631.139

**Published:** 2025-01-29

**Authors:** Nabila Shaikh, Robertus van Aalst, Rebecca C Harris, Clotilde El Guerche-Séblain

**Affiliations:** Sanofi, Reading, England, United Kingdom; Sanofi Vaccines, Lyon, Auvergne, France; Sanofi, Reading, England, United Kingdom; Sanofi vaccines, LYON, Rhone-Alpes, France

## Abstract

**Background:**

The emergence of SARS-CoV-2 has profoundly impacted global healthcare systems, raising concerns about its interaction with seasonal influenza and the appropriateness of concomitant vaccination campaigns. This study aims to compare the burden and timing of COVID-19 and influenza-associated hospitalizations during the post-pandemic period.

**Figure 1:**
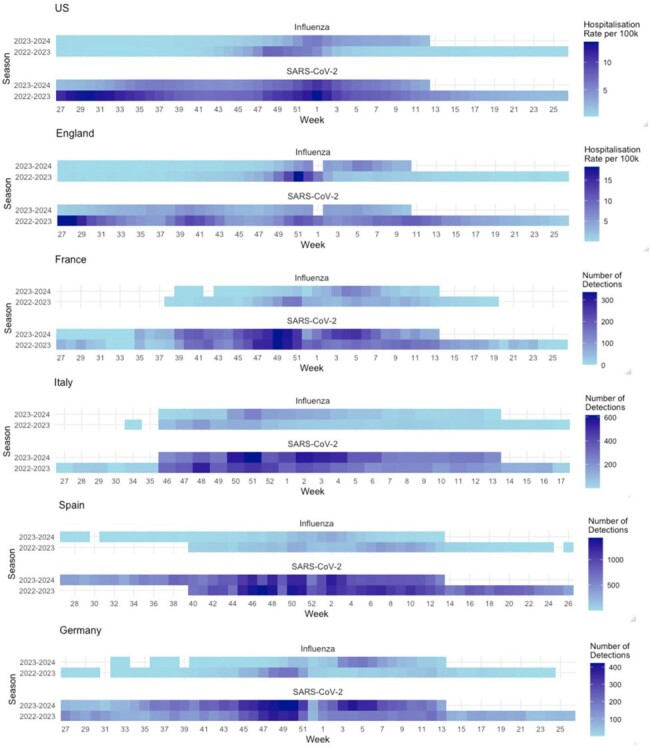
Evolution of Influenza and Covid-19 activity by season over 2022-2023 and 2023-2024

**Methods:**

We retrospectively analyzed laboratory-confirmed hospitalization data from sentinel surveillance systems for influenza and COVID-19 across two seasons (2022-2023 to 2023-2024) in England, the US, and four European countries (France, Germany, Italy, Spain). We examined absolute hospitalization numbers in the EU, weekly rates in England, and both rates and numbers in the US to identify seasonal peaks and describe epidemiological trends.

Annual cumulative number of laboratory-confirmed hospitalizations in Germany, Spain and US by season (annual period defined as epi week 27 – epi week 26)

**Figure:**
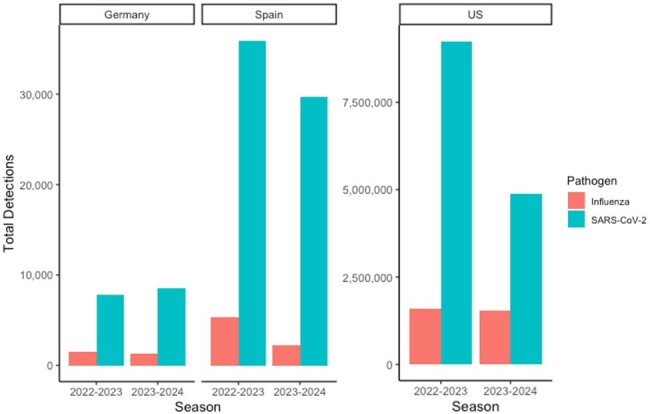

**Results:**

Peak laboratory-confirmed hospitalizations in EU countries for COVID were higher than for influenza, ranging from 0 to 1,427 per week and 0 to 366 per week, respectively. Similarly in the US, hospitalizations for COVID-19 and influenza varied from 44,760 to 319,445 and 3,301 to 172,784, respectively. England had higher peak weekly rates for both influenza (18.1 per 100,000) and COVID (18.3 per 100,000) compared to the US (7.4 per 100,000 for influenza and 13.7 per 100,000 for COVID).

Across the EU, England, and the US, peak influenza activity occurred between Week 47 and Week 6 in both 2022-2023 and 2023-2024 seasons. While COVID-19 activity peaked alongside influenza, activity remained high beyond the winter seasons.

Annual cumulative hospitalizations for COVID-19 were 4, 5, and 12 times higher than influenza in the US, Germany, and Spain, respectively. Cumulative hospitalizations for other countries were not assessed due to missing data.

**Conclusion:**

COVID-19 and influenza continue to pose a significant burden on healthcare systems. While differing testing practices between viruses and between countries introduces limitations to this analysis, our findings confirm that COVID-19 circulation is not restricted to winter seasonality, and surveillance and prevention measures should be reinforced year-round. Further research is also required to properly inform the validity of concomitant vaccination policy.

**Disclosures:**

**Nabila Shaikh, MSc**, Sanofi: Stocks/Bonds (Public Company) **Robertus van Aalst, PhD, MSc**, Sanofi: Employee|Sanofi: Stocks/Bonds (Public Company) **Rebecca C Harris, MBioch, MSc, PhD**, Sanofi vaccines: Stocks/Bonds (Private Company) **Clotilde El Guerche-Séblain, PhD**, Sanofi vaccines: Stocks/Bonds (Private Company)

